# Progress in the development of an Advax-adjuvanted protein capsular matrix vaccine against typhoid fever

**DOI:** 10.36922/mi.4497

**Published:** 2024-10-04

**Authors:** Nikolai Petrovsky, Kevin P. Killeen

**Affiliations:** 1 Vaxine Pty Ltd, 11-13 Walkley Avenue, Warradale, South Australia, Australia; 2 Matrivax Research and Development Corporation, Boston, Massachusetts, United States of America

**Keywords:** Vaccine, Typhoid, Adjuvant, Immunization, Advax

## Abstract

Typhoid fever, caused by *Salmonella* Typhi, remains a significant global public health concern, with an estimated 11 – 20 million cases annually. Vaccines are critical to controlling typhoid fever. Widespread vaccination diminishes the emergence of antibiotic-resistant strains of *S.* Typhi. The economic benefits of vaccination are also substantial, as the costs of treating typhoid fever and its complications can be significant. Ty21a^®^, a killed whole-cell vaccine, and Vivotif^®^, a live-attenuated vaccine, have been available for decades but have relatively short durations of action and only provide partial protection. Vi polysaccharide-conjugate vaccines have improved the durability of protection, but there is still room for improvement. Typhax^™^, a novel alternative to traditional conjugate vaccines, utilizes Vi polysaccharide that is non-covalently entrapped in a poly-L-lysine and CRM197 protein matrix crosslinked by glutaraldehyde. When formulated with Advax-CpG^™^ adjuvant, Typhax demonstrated promising results in a range of animal models including mice, rabbits, and non-human primates in which it induces high and sustained serum anti-Vi immunoglobulin G and serum bactericidal activity, without any safety or reactogenicity issues. This novel vaccine approach offers the potential for a low-cost, more effective, and durable vaccine against typhoid fever, avoiding the need for frequent booster doses.

## 1. Introduction

Typhoid fever, caused by *Salmonella enterica* serovar Typhi (*S.* Typhi), is transmitted to humans through contaminated food and water, and it remains a major cause of pediatric deaths in rural regions of developing countries.^1^ According to estimates, there were 14.3 million cases of typhoid and paratyphoid fevers globally in 2017.^2^ Immunocompromised people are particularly susceptible to infection with *S.* Typhi and are at increased risk of developing severe disease. *S.* Typhi, a Gram-negative rod, possesses several virulence factors that enable it to survive the host’s anti-bacterial response, including toxins and metallophores essential for the bacterium’s survival.^3^

Although prompt antibiotic therapy can decrease the severity, duration, complications, and mortality of typhoid fever, *S.* Typhi has acquired resistance to oral antibiotics widely available in recent years.^4^ Approximately 1 – 4% of patients chronically harbor *S.* Typhi in their intestinal tract and gall bladder and act as asymptomatic carriers. With increasing antibiotic resistance and slow progress in improving water and sanitation in many developing countries, vaccination against *S.* Typhi is the most effective means of reducing typhoid fever deaths.^5^ An episode of typhoid fever typically results in lifelong protective immunity, with both cell-mediated and humoral immunity being elicited following infection.^6^ At present, there are three major types of vaccines commercially available for typhoid prevention: oral live-attenuated vaccines, parenterally administered unconjugated Vi polysaccharide, and polysaccharide-protein conjugates (summarized in Table 1).

## 2. Live-attenuated oral vaccines

The Vivotif^®^ vaccine is an orally administered vaccine that is based on the attenuated S. Typhi Ty21a strain in which multiple pathogenicity-associated genes have been mutated, including those mediating the production of the Vi polysaccharide. Three doses of Ty21a administered in enteric-coated capsules as an every other day regimen were shown in one study to confer 67% protection over 3 years and 62% protection over 7 years.^7^ However, this formulation is impractical for infants and toddlers and is only recommended for children over 6 years of age. Of concern, a recent human challenge study revealed no protective efficacy following the live-attenuated oral vaccine regimen with the Ty21a vaccine failing to induce an increase in anti-Vi antibody levels.^8^ Pre-existing anti-Vi antibody levels were seen in those study subjects who showed protection, consistent with anti-Vi antibody playing a role in *S.* Typhi protection. Another downside of live-attenuated vaccines is that they are associated with frequent gastrointestinal side effects due to causing an attenuated typhoid-like illness. Live vaccines are contraindicated in those with primary or acquired immunosuppression in whom they could cause severe typhoid disease. Being an older technology, live vaccines also may contain animal-derived products such as bovine collagen which are no longer contained in newer vaccine types.

### 2.1. Vi polysaccharide subunit vaccines

Vi subunit vaccines (Typhim Vi^®^, Typherix^®^, and Typbar^®^) are single-dose, intramuscularly administered polysaccharide vaccines approved for use in adults and children >2 years of age. Polysaccharide-based vaccines confer variable and short-lived immunity. Estimates of vaccine efficacy of around 50% have been found in areas where typhoid fever is endemic, and evidence of indirect protection of unvaccinated neighbors of vaccinees has been found.^9,10^ Immunity wanes within 2-year post-vaccination and there is no evidence that protective efficacy lasts beyond 3 years. Re-vaccination every 2 years is recommended for U.S. travelers to *S.* Typhi endemic areas. Thus, the widely available Vi polysaccharide subunit vaccines confer relatively short-term protection against typhoid in older children and adults and are poorly immunogenic in infants under 2 years of age due to their inability to elicit a T-cell-dependent immune response.^11^ This limits the utility of the pure polysaccharide vaccines for *S.* Typhi eradication campaigns.

### 2.2. Vi polysaccharide-conjugate vaccines

Vi polysaccharide-conjugate vaccines in which the Vi polysaccharide is covalently coupled to a protein antigen have recently been developed and shown to be highly effective in children as young as 3 months of age.^12^ Immunization with a 25 μg dose of a Vi-conjugate vaccine using CRM197 as a carrier protein developed by the Novartis Vaccine Institute for Global Health 28 days achieved anti-Vi geometric mean titer (GMT) of 304 EU/mL. Bharat Biotech’s Vi-conjugate vaccine (Typbar-TCV^™^), when administered to human children at a 25 μg dose, achieved anti-Vi GMTs approximately 3-fold higher than those obtained with their equivalent unconjugated polysaccharide vaccine (Typbar^™^). Two years after vaccination, anti-Vi titers in those receiving the conjugate vaccine remained almost 2-fold higher (GMT 82) than in those that received the polysaccharide vaccine (GMT 46).^13^ The International Vaccine Institute reported a phase 2 study of a diphtheria toxoid-conjugated Vi vaccine in children aged 6 – 24 months which achieved anti-Vi GMT of 444.38 EU/mL.^14^ In Vietnam, Vi polysaccharide conjugated with recombinant *Pseudomonas aeruginosa* exotoxin A conferred >90% protection against typhoid over the first 27 months and >80% over 46 months.^15^ From this study, it was estimated that the protective level of anti-Vi immunoglobulin G (IgG) is 3.5 EU/mL. A phase 1 study of the Vi-diphtheria toxoid conjugate (Vi-DT) conducted in the Philippines enrolled subjects aged 2 – 45 years who received either Vi-DT or Typhim Vi vaccine.^16^ The conjugated Vi-DT vaccine generated a 4-fold higher Vi GMT compared to the pure polysaccharide Typhim Vi vaccine. Similarly, in a phase 1 study in European adults, anti-Vi GMT levels 4-week post-vaccination in the Vi-CRM197 group (304 EU/mL) were 6 times higher than in those vaccinated with Typhim Vi (52 EU/mL).^17^

Collectively, Vi-conjugate vaccines elicit approximately 3 – 6-fold higher peak anti-Vi antibody levels than pure polysaccharide vaccines and thereby provide more durable protection. For example, the conjugated typhoid vaccine manufactured in India is said to provide 5 years of protection when used in typhoid-endemic regions. It may be able to achieve this duration of protection because, in endemic regions, periodic re-exposure to *S.* Typhi provides regular boosting to Vi antibody levels, thereby helping maintain titers above protective levels for an extended timeframe. Travelers from countries where typhoid is not endemic would not get the benefit of such periodic endemic re-boosting, meaning that conjugate vaccines may provide a much shorter duration of protection to those living outside of endemic areas.^18^ Hence, there remains a need to create more potent and durable typhoid vaccines.

## 3. Potential next-generation typhoid vaccine approaches

Even with the recent advent of the conjugate vaccines, the unmet need for more effective and durable typhoid vaccines remains. How might this be achieved? One area might be to explore modified polysaccharide antigens better able to present key neutralizing epitopes so as to maximally stimulate memory B cell responses.^19^ Consideration could be given to including additional antigens, such as *S.* Typhi lipopolysaccharide (LPS) antigens, in the vaccine.^20^ The antigens could be formulated with newer, more potent adjuvants.^21^ Finally, vaccine delivery approaches to better stimulate mucosal immunity could be attempted.^22^ While mRNA approaches have been touted as a way forward for many other traditional vaccines, these can only encode protein antigens and hence are not currently an option to replace vaccines where non-protein antigens such as polysaccharides are involved.^23^ Similarly, while viral-like particles could theoretically be used as the protein carrier on which to conjugate polysaccharide antigens, little work has been done in this area, presumably due to the additional complexity of conjugating polysaccharides to the particles as opposed to conjugating them to soluble proteins.

**Figure 1.**
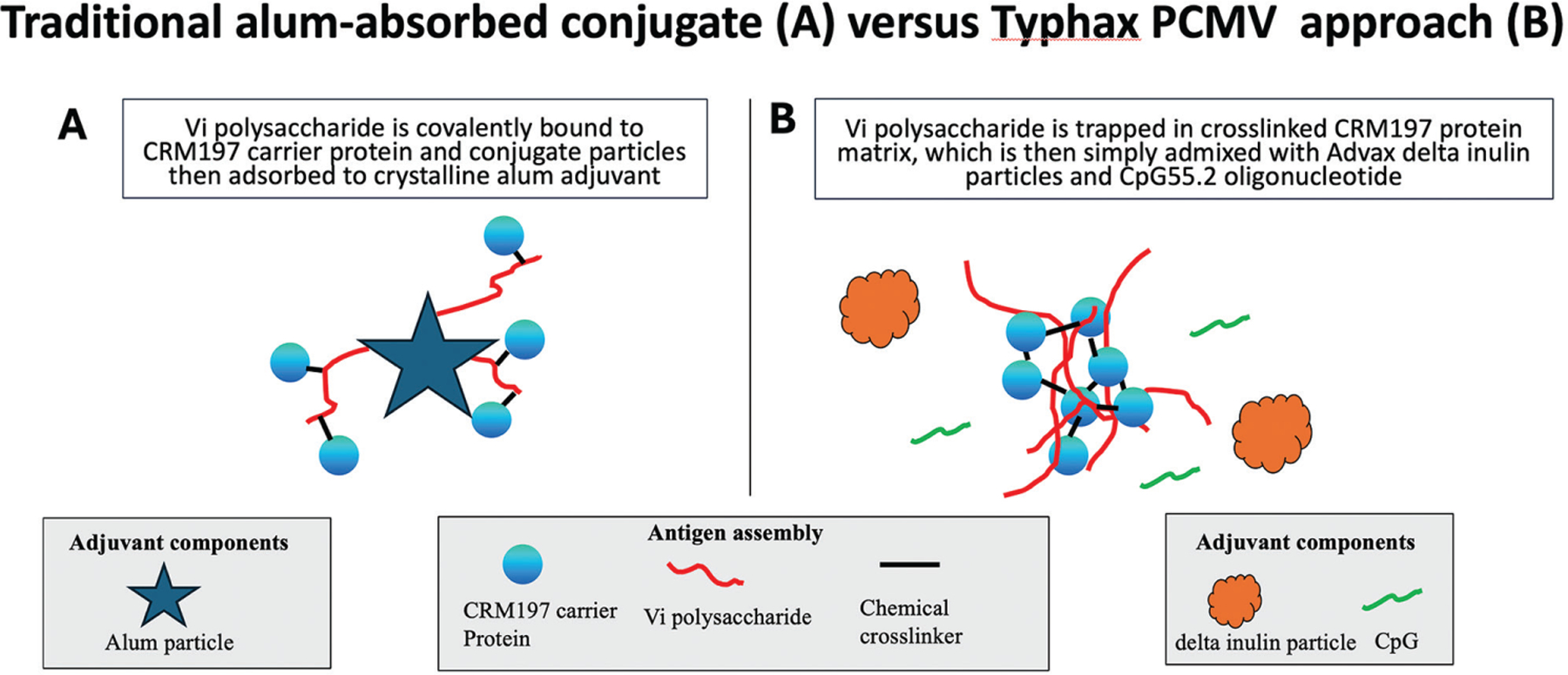
Schematic shows the differences between the traditional polysaccharide conjugation approaches where short segments of polysaccharide are directly crosslinked to the carrier protein and the protein capsular matrix vaccine (PCMV) approach where the carrier protein is crosslinked to itself, thereby trapping the full-length polysaccharide chains in the crosslinked protein matrix. The PCMV antigen can then be formulated with a relevant adjuvant such as Advax-CpG^®^ to further enhance vaccine immunogenicity

An example of an alternative to traditional polysaccharide-conjugate approaches is the protein capsular matrix vaccine (PCMV) approach.^19^ The PCMV process non-covalently entraps polysaccharide antigens in a crosslinked protein matrix (depicted in Figure 1A). The PCMV process is simpler and cheaper than the manufacture of polysaccharide-conjugate vaccines and allows full-length bacterial polysaccharides to be used, whereas in conjugate vaccines typically only short pieces of the polysaccharides are used. In the PCMV process, the Vi polysaccharide antigen purified from *S.* Typhi is entrapped in a glutaraldehyde-catalyzed matrix of crosslinked α-poly-L-lysine (α-PLL) and CRM197 protein, a genetic toxoid of diphtheria toxin and a common carrier protein used in conjugate vaccines. The non-covalent entrapment of polysaccharide antigens in a crosslinked matrix of protein provides the benefits of conjugated vaccines such as the ability to induce helper T cells and enhance antibody levels, without the complexity and expense of direct multi-step polysaccharide conjugation to a carrier protein.^19^ Notably, PCMV was effective in inducing helper T cells for the B cell response to the polysaccharide antigen, showcasing all the benefits of a conjugate vaccine.^24^ PCMV technology thereby offers a simpler and cheaper means to manufacture the *S.* Typhi vaccines with similar characteristics as the conjugate vaccine, an important consideration given that the vast majority of *S.* Typhi vaccines are needed in the poorest developing countries where *S.* Typhi is endemic. Such cheap and affordable vaccines, particularly if they provide long-term durable protection, could be extremely important for global *S.* Typhi eradication campaigns. The PCMV approach has already been shown to be safe and effective in a human phase 1 clinical trial of Typhax^™^.^25^

## 4. Typhoid vaccine adjuvants

Pure polysaccharide vaccines typically are T-cell independent and hence unlikely to benefit from formulation with traditional adjuvants. By contrast, protein-conjugate vaccines are able to enlist helper T cell responses directed at the carrier protein, with these T cells then able to provide help to polysaccharide-specific memory B cells to become long-lived plasma cells.^12^ The PCMV approach allows the use of an adjuvant to further enhance vaccine potency (Table 2). To date, commercial polysaccharide-conjugate vaccines, such as Prevnar, have either been used alone or with aluminum salt adjuvants.^26^ Advax^®^ adjuvant (VO_0005324^1^) was developed as part of the NIH Adjuvant Development Program.^27^ and is derived from inulin polysaccharide formulated into microcrystalline particles referred to as delta inulin.^28–30^ Advax^®^ adjuvants have been demonstrated to enhance immunogenicity and vaccine protection across a diversity of viral, bacterial, and parasitic vaccines.^31–36^ Advax^®^ formulations can be complemented by the addition of TLR9-active CpG oligonucleotides to form a combination adjuvant known as Advax-CpG (VO_0005207^2^) that further enhances vaccine potency.^31,32,37^ CpG55.2 is a potent human TLR9 agonist that was the first licensed human drug molecule designed by artificial intelligence. Advax^®^ adjuvants were shown to be safe and well-tolerated and enhance immunogenicity in human clinical trials of influenza, hepatitis B, and insect sting allergy vaccines^38–40^ and are a key component in SpikoGen^®^ vaccine, a recombinant protein COVID-19 vaccine licensed for use in the Middle East in adults and children aged 5 years and older, with 8 million doses having been safely delivered.^41–44^ One of the notable properties of Advax^®^ adjuvants is that they are highly effective in newborns where they uniquely overcome neonatal immune hypo-responsiveness.^45–47^ This makes the Advax^®^ family of polysaccharide adjuvants uniquely suited for development with *S.* Typhi vaccines.

## 5. Advax-CpG adjuvanted Typhax vaccine

Immunizations of mice, rabbits, and non-human primates (NHP) with the Typhax^™^ vaccine formulated with Advax-CpG adjuvant elicited anti-Vi IgG responses up to 1,000-fold higher than those induced by an equivalent dose of the commercial Typhim Vi vaccine.^24^ Notably, the Advax-CpG adjuvanted Typhax vaccine did not induce polysaccharide-associated immune suppression^48^ with the anti-Vi IgG levels increasing after each booster immunization. Human data showed Vi antibodies induced by Typhim Vi immunization decay back to baseline by 24 months post-immunization.^49^ By contrast, in immunized macaques the anti-Vi IgG responses induced by Advax-CpG adjuvanted Typhax vaccine were durable and remained well above baseline levels up to 9 months post-immunization.^24^ Based on the estimated protective level of anti-Vi IgG of 3.5 EU/mL,^15^ the antibodies induced by Advax-CpG adjuvanted Typhax seem likely to remain well in excess of this level for an extended period that may last many years, thereby avoiding the need for regular boosters every 1 – 2 years. The exceptionally high Vi antibody levels induced by Advax-CpG adjuvanted Typhax vaccine indicate that although the Vi polysaccharide is not conjugated to the CRM197 carrier protein, the Typhax vaccine is working in a T cell-dependent manner. This was confirmed by data showing that the anti-Vi response induced by Advax-CpG adjuvanted Typhax vaccine was markedly attenuated in CD4 T cell-depleted mice, thereby confirming the response to be T cell-dependent.^24^ Notably, in NHP, Advax-CpG adjuvanted Typhax vaccine achieved peak anti-Vi responses that were approximately 25 times those achieved by Typhim Vi.^24^

## 6. Role of serum bactericidal activity (SBA) in typhoid vaccines

SBA has been found to be a strong correlate of protection for bacterial vaccines including those targeted at cholera and pneumococcal and meningococcal diseases.^50,51^ SBA has also been shown to be inversely correlated with susceptibility to typhoid fever. Anti-Vi antibody and SBA levels were not correlated in individuals exposed to natural infection with *S.* Typhi in an endemic area.^52^ This suggests the main protective *S.* Typhi antibody responses induced by natural infection or oral vaccines may be directed against *S.* Typhi LPS, rather than Vi polysaccharide. Vi-DT contains a small amount of endotoxin (9.65 EU/dose) which is within the acceptable range for human vaccines. Hence, residual *S.* Typhi LPS contaminating Vi and Vi-DT vaccines may act as an antigen and be responsible for inducing antibodies with SBA in vaccinated individuals.^16^

Some studies suggest that anti-Vi IgG contributes to reduced disease symptoms and prevention of *S.* Typhi infection.^8,53^ Bactericidal antibody induced by an oral attenuated vaccine reduced typhoid severity but did not protect against clinical disease in a human challenge model.^20^ The bactericidal activity was attributed to anti-LPS antibodies rather than anti-Vi antibodies, and the depletion of LPS antibodies significantly reduced bactericidal activity.^20^ The Advax-CpG adjuvanted Typhax vaccine group induced high SBA in addition to high anti-Vi IgG.^24^ It is possible the SBA was due to anti-LPS antibodies induced by small amounts of bacterial LPS contaminating the *S.* Typhi polysaccharide used to make Typhax. Notably, a human phase 1 study of a Vi-conjugate vaccine showed only a weak correlation between anti-Vi and SBA titer post-immunization with a 6-fold increase post-immunization in SBA in the Vi-conjugate vaccine group and a 4-fold increase in SBA in the Typhim Vi group.^16^ In a previous study, Advax-CpG adjuvanted Typhax vaccine drove a 50-fold increase in SBA post-immunization as compared to the Typhim Vi group which induced only a short-lived 2-fold increase in SBA.^24^ Human typhoid challenge studies may offer a faster and less expensive way to confirm Typhax vaccine efficacy as compared to traditional large phase 3 outcome studies that seek to assess vaccine impact on spontaneous natural infections.^54^

## 7. Future directions

Parenteral typhoid fever vaccines have evolved from pure Vi polysaccharide vaccines to protein-conjugated Vi vaccines that provide better helper T-cell response and result in higher and more durable Vi antibody levels, thereby extending protection. The application of conjugation technology to typhoid vaccines represented a significant advance over unconjugated polysaccharide vaccines, with modest improvements in efficacy and durability, although at an increased cost due to the complexities of polysaccharide conjugation chemistry. A further potential advantage of protein conjugates is that they can benefit from an adjuvant to further increase their potency. In a major step forward, PCMV technology was used to create the Typhax vaccine, a simpler and cheaper approach to polysaccharide conjugation whereby the polysaccharide is trapped in a protein matrix rather than directly conjugated to the carrier protein. This achieves all the benefits of a polysaccharide-conjugate vaccine without involving complex procedures and high costs. To further build on this platform, we were able to show that the PCMV-based Typhax vaccine can be conveniently combined with Advax-CpG adjuvant, thereby delivering a typhoid vaccine of unmatched potency, which for the 1^st^ time has the potential to provide long-term durable protection against *S.* Typhi without the need for regular boosters. This could further raise the bar for typhoid vaccine efficacy and durability. An Advax-CpG adjuvanted Typhax vaccine could make typhoid vaccines more accessible for poor endemic countries such as Nepal by reducing manufacturing costs and reducing the need for regular booster doses. This might thereby help bring the world one-step closer to typhoid eradication. While Typhax PCMV antigen and Advax-CpG55.2 adjuvant have separately been shown to be safe and effective in human testing, what is now needed is a human trial of the combination of these two complementary technologies, with the potential to deliver a typhoid vaccine of unmatched safety, efficacy, and durability.

## 8. Conclusion

Advax-CpG55.2-adjuvanted Typhax induced high and sustained serum bactericidal activity against S. Typhi in mice, rabbits and non-human primates. It is thereby a highly promising vaccine development candidate to provide robust and durable protection against typhoid fever.

## Figures and Tables

**Table 1.** The pros and cons of typhoid vaccine approaches

Vaccine type	Brands	Pros	Cons
Live attenuated *Salmonella Typhi* strain Ty21a	Vivotif oral	• Relatively low cost	• Requires 3 doses • Only indicated in children aged >6 years • Contraindicated in primary and acquired immunodeficiency • Cannot be given at the same time as antibiotics • Frequent gastrointestinal side effects • Contains bovine-derived material • May have weak or no efficacy • Cannot be adjuvanted because it is a live vaccine
Pure Vi polysaccharide vaccine	Typhim Vi^®^, Typherix^®^, and Typbar^®^	• High safety • Only a single dose is required	• Weak efficacy at ~50% • Only indicated in infants aged >2 years of age • Short duration of protection (<2 years) • Unable to boost response • Cannot be adjuvanted as it is T-cell independent
Vi polysaccharide conjugate vaccine	Typbar-TCV^™^	• High safety • More durable protection • Effective in children aged >3 months • 3- to 6-fold higher peak anti-Vi responses • Can potentially be adjuvanted	• More expensive • Protection would wane after 5 years
Protein capsular matrix vaccine	Typhax/Advax-CpG^™^	• High safety • Up to 1000-fold higher peak anti-Vi responses, suggesting the possibility of long-term protection • Inclusion of Advax-CpG adjuvant overcomes polysaccharide-associated immune suppression • Anti-Vi antibodies able to be strongly boosted with repeated doses • Induces serum bactericidal antibodies • Low cost	• None

**Table 2.** The pros and cons of potential typhoid vaccine adjuvants

Adjuvant	Tradenames	Pros	Cons
Aluminum salts (Alum)	AdjuPhos^®^, Alhydrogel^®^	• Low cost • Easy to formulate • Enhances anti-Vi response	• Imparts major Th2 immune bias which may predispose to allergy/anaphylaxis • Does not overcome polysaccharide-associated immune suppression • Only modest enhancement of Vi antibody titers • Low efficacy in children aged <3 months
Delta inulin-CpG oligonucleotide combination adjuvant	Advax-CpG^™^	• Low cost • Easy to formulate • Overcomes polysaccharide-associated immune suppression • Strong enhancement of anti-Vi response • Induces production of serum bactericidal antibodies • Effectively overcomes neonatal immune hypo-responsiveness in newborns	• None

## Footnotes

1
https://vac.niaid.nih.gov/view?id=38

2
https://vac.niaid.nih.gov/view?id=37
